# Prognostic value of downregulated 5-hydroxymethylcytosine expression in renal cell carcinoma: a 10 year follow-up retrospective study

**DOI:** 10.7150/jca.38283

**Published:** 2020-01-01

**Authors:** Song Chen, Qiang Zhou, Tongzu Liu, Weibing Zhang, Xian-Tao Zeng, Zhongqiang Guo

**Affiliations:** 1 Department of Urology, Zhongnan Hospital of Wuhan University, Wuhan 430071, China.; 2 Center for Evidence-Based and Translational Medicine, Zhongnan Hospital of Wuhan University, Wuhan 430071, China.

**Keywords:** renal cell carcinoma, 5-hydroxymethylcytosine, clinical significance, nomogram.

## Abstract

5-hydroxymethylcytosine (5hmC) is converted from DNA methylation of cytosine (5mC) by the catalysis of TET proteins, and proposed to be involved in tumorigenesis. However, the prognostic value of 5hmC in renal cell carcinoma (RCC) is still unclear. This study aimed to define the clinical significance of 5hmC in RCC. We performed dot blot assays to measure the relative expression of 5hmC in RCC. We reviewed the clinical records of 310 RCC patients and performed immunohistochemical (IHC) staining of 5hmC. The overall survival (OS) and cancer specific survival (CSS) of all patients were recorded over a 10-year follow-up period. Effective prognostic nomograms which contained 5hmC were established to provide individualized OS and CSS in RCC. 5hmC expression level was significantly decreased in RCC tissues compared with those in the normal counterparts. Kaplan-Meier curves revealed that high 5hmC expression had a good prognostic impact on RCC patients. Cox multivariate survival analyses further indicated 5hmC was an independent prognostic factor for RCC survival. Nomograms constructed based on cox regression analysis were available to calculate the survival probability directly. Calibration curves displayed good agreements. The findings were validated with an independent external cohort included 77 RCC cases. Thus, we believe we have found a significative prognostic factor for RCC.

## Introduction

Renal cell carcinoma (RCC) is a malignant tumor mainly originating from renal tubular epithelial cells, accounting for 2-3% of adult malignant tumors. Currently, the incidence rate and the mortality rate are increasing in worldwide 1-2. Patients with early stage RCC have no obvious clinical symptoms, and they often have progressed to the advanced stage when obvious discomfort occurs. More specifically, RCC is a common group of chemotherapy-resistant diseases, which are not sensitive to traditional chemoradiotherapy or hormone therapy 3-4. At present, the most common treatment for RCC is radical nephrectomy or nephron sparing partial nephrectomy. However, about 30% of patients have metastasized cancer when diagnosed, and about half of the patients who have not metastasized will experience recurrence or metastasis after radical resection 5-6.

The clear cell renal cell carcinoma (ccRCC) is the primary type of RCC, which represents 75%-80% of all RCCs 7. Some studies have shown that patients with ccRCC have a poor prognosis. The 5-year survival rate of ccRCC patients is about 60%, which is lower than other types of RCC patients 8-9. Currently, there are many clinical factors used to evaluate the prognosis of RCC, including histological type, Fuhrman's nuclear grade, tumor stage, tumor necrosis, lymph node metastasis and vein invasion. However, a single clinical factor is not ideal for predicting the prognosis of RCC 10-11. Therefore, prognostic monitoring of RCC and more effective targeted therapies are essential to reduce patient mortality and improve cure rate.

There are exact evidences that the Von Hippel-Lindau (VHL) tumor suppressor gene is mutated or inactivated in more than 80% of ccRCC patients. However, in mouse (Mus musculus) experiments, it was found that the deletion of this gene did not cause the formation of clear cell carcinoma, which indicates that there are other tumorigenic mechanisms 12-13. Previous studies have also found that mutations associated with renal carcinogenesis and prognosis occur mainly in genes encoding epiregulatory factors, such as the BAP1 gene that regulates histone H2A ubiquitination, the histone methyltransferase gene SETD2, and the TET2 gene which catalyzes the conversion of DNA methylation of cytosine (5mC) to 5hmC 14-15.

As one of the most widely studied epigenetic modifications, 5mC of the CpG dinucleotide in gene promoter is usually related to the transcriptional silencing of cancer cells 16. Several important studies showed DNA methyltransferases could catalyze and maintain the transformation of cytosine to methyl cytosine, but the Ten-Eleven-Translocation (TET) family of TET1, TET2 and TET3 mediate the reverse process 17-19. Gradually, these TET proteins mediate the transformation of 5mC to 5hmC, 5-formyl cytosine and 5-carboxyl cytosine 20.

Emerging evidence indicates that 5hmC may also act a role of stable epigenetic marker with incomplete characterization 21. In our previous study, we found that almost all patients with RCC have a loss of 5hmC, which is considered to be a result of the downregulation of isocitrate dehydrogenase 1 (IDH1) 22. Nevertheless, the prognostic value of 5hmC in RCC is still unclear. In present study, we attempted to define the clinical significance of 5hmC in RCC (including ccRCC and other RCC).

## Material and methods

### Study Patients

This study contained a development cohort and a validation cohort. The development cohort included 310 RCC patients at the Department of Urology, Zhongnan Hospital of Wuhan University from January 2007 to March 2015. All patients had undergone surgery treatment meanwhile surgical tissue specimens was gathered. Dot blot assays and IHC staining of 5hmC were performed in these surgical tissue specimens. Clinical, pathological, follow-up data record was collected. The analyzed clinical and pathological data consisted of age, gender, tumor size, capsule invasion, vein invasion, tumor necrosis, TNM stage, clinical stage, Furhman grade and 5hmC level. The follow-up data included OS and CSS of RCC. All patients provided the informed consent. The Ethics Committee at Zhongnan Hospital of Wuhan University has approved the using clinical information and surgical tissue specimens in our study (approval number: 2015029). All procedures and ethical standards were done in accordance with the national research committee and/or institutional. The validation cohort was a tissue microarrays (TMA, ID: KIC387) purchased from Guilin Fanpu Biotech Co., Ltd, which included 77 RCC patients from July 2006 to October 2009.

### Inclusion criteria

Patients were enrolled in this study if they met all the following criteria: (i) presence of primary RCC; (ii) without any a prior history of preoperative target therapy, chemoradiotherapy or hormone therapy; (iii) underwent radical nephrectomy or nephron sparing partial nephrectomy; (iv) surgical tissue specimens (tumor/normal) were collected; (v) had a complete and detailed clinical, pathological, follow-up data record.

### Exclusion criteria

Patients meeting any of the following criteria were excluded: (i) presence of metastatic/secondary tumor of the kidney; (ii) any prior history of preoperative targeted therapy, chemoradiotherapy or hormone therapy; (iii) patients who did not undergo surgery; (iv) surgical tissue specimens were unavailable; (v) any incomplete clinical, pathological or follow-up data.

### Cell lines

RCC cell lines (Ketr-3, OS-RC-2, 769-P and 786-O) were cultured in RPMI1640 medium (Gibco, China) supplemented with 10% FBS. Human renal proximal tubular epithelial cell line (HK-2) was maintained in KSF medium with epidermal growth factor as well as bovine pituitary extract (Gibco, Carlsbad, CA, USA). All cell lines were purchased from the Stem Cell Bank, Chinese Academy of Sciences in Shanghai, China. These cell lines were grown at 5% CO_2_, 37℃ in a humidified incubator (Thermo Scientific).

### Cell lines and tissue genomic DNA extraction

A proportion of the surgical tissue specimens were cryopreserved at -80℃. We used tumor as well as the matched normal tissue from the same radical nephrectomy patients for DNA analysis. Genomic DNA was isolated from cultured RCC cell lines and surgical tissue specimens with the Qiagen DNA Mini Kit (250) (Qiagen, Cat#: 51306).

### Quantitative 5hmC analysis

Quantitative 5hmC analysis performed via DNA dot blotting. Genomic DNA was heated for deformation and then chilled; temperature/time were 95℃/10min and 4℃/5min, respectively. The DNA samples were dried on wet Hybrid Membrane. The samples were then spotted on positively charged membranes and afterwards serially diluted in NaOH/EDTA solution. The membrane was blocked with 5% non-fat milk in TBST for 1 h at room temperature (RT), followed by incubation with primary antibody against 5hmC (dilution 1:5000, Active motif) overnight at 4℃. Anti-rabbit IgG-HRP antibody was incubated with the membrane for 1 h at RT. At last, the membrane was treated with ECL Kit (GE Amersham, Cat#: RPN2232) after washing three times with TBST.

### Immunohistochemical staining and scoring

A proportion of surgical tissue specimens were fixed with formalin to for paraffin-embedded. IHC analyses was performed on 4 μm thick sections. Briefly, each slide was incubated with primary antibody against 5hmC (1: 5000, Active motif, Cat#: 39999) overnight after a series of procedures (de-paraffin, antigen retrieval, rinse). This was followed by an incubation with the anti-rabbit IgG-HRP antibody (EnVision Dual Link, Dako) for 30 min. The membrane was then washed five times with TBST and enriched with the brown color of DAB Enhancer (Dako). The 5hmC expression was evaluated by three experienced pathologists. Receiver operating characteristic (ROC) curve was generated for 5hmC expression level to calculate the areas under the curve (AUC). The highest Youden's index, as the optimized point, was used to determine the optimal cut-off value of 5hmC expression level based on the ROC curve.

### Statistical analysis

All continuous measures were compared by two-sample *t* test, graded variables were analyzed with Mann-Whitney test. The associations between 5hmC expression level and specific clinicopathological factors in RCC patients (including ccRCC and other RCC) were analyzed with Chi-square test. Kaplan-Meier curves were generated to estimate OS and CSS, and the log-rank test was used to assess survival differences among subgroups. Cox univariate and multivariate survival analyses were used to estimate the independent factors of survival rate. Nomograms were generated based on cox regression analyses. The calibration curves were generated to assess the agreements of the nomogram-predicted probability with the actual observed probability. The stability (sensitivity and specificity) of the prediction nomograms were validated with the independent external cohort. We used SPSS 16.0 and GraphPad Prism 7 to perform all statistical analyses. Nomograms and calibration curves were generated with R version 3.5.0 and a p value <0.05 was considered statistically significant.

## Results

### 5hmC level was significantly decreased in RCC

**Figure 1:**
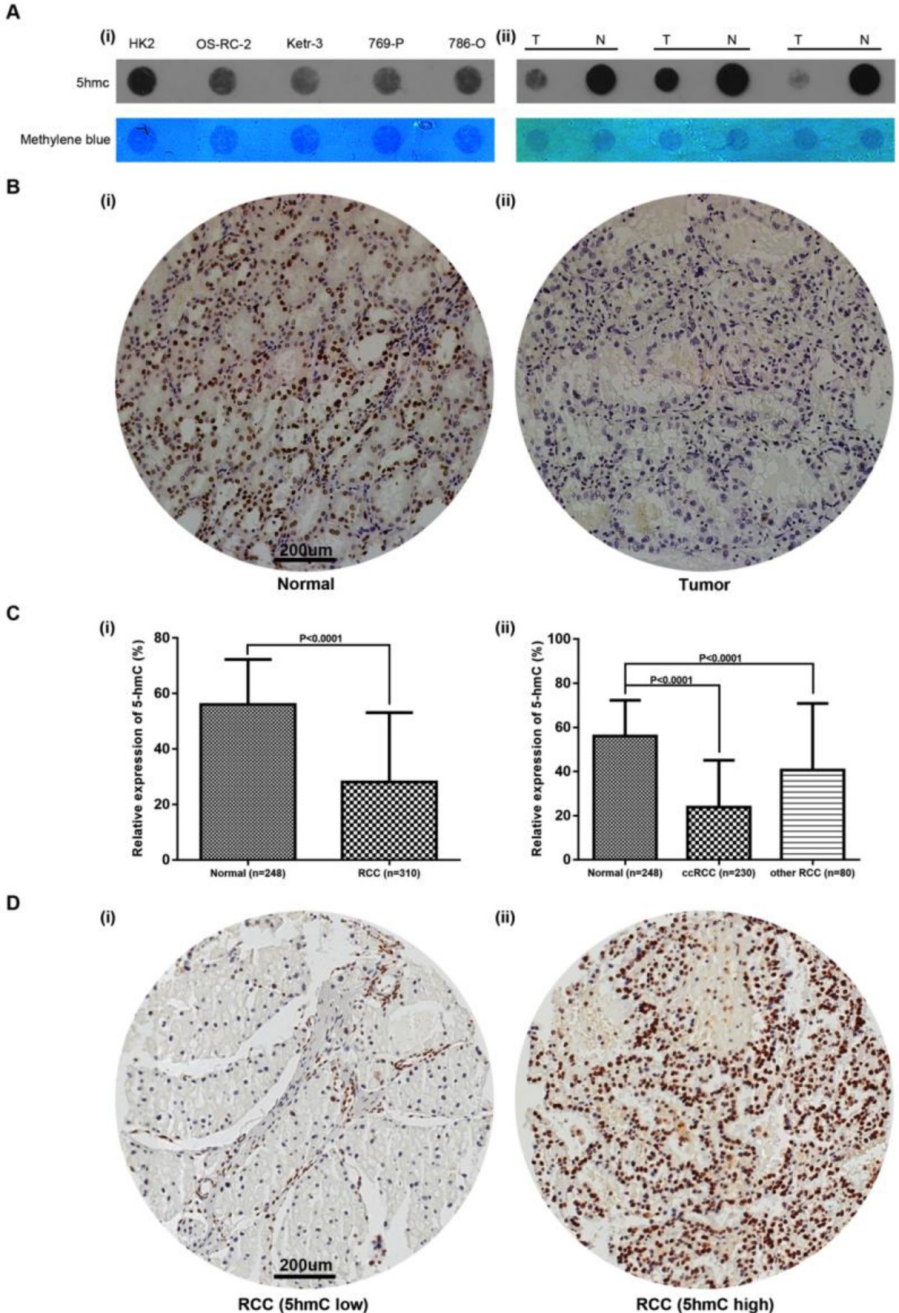
** Relative expression of 5hmC in RCC (A)** Dot blot assays of 5hmC in RCC cell lines/HK-2 as well as paired RCC tissues/renal tissues of three RCC patients. Equal loading was validated by methylene blue staining. T, tumor; N, matched normal tissue; **(B)** A representative IHC staining of 5hmC in tumor and paired normal samples of RCC patients. Scare bar, 200 μm; **(C)** Relative expression of 5hmC level in RCC (include ccRCC and other RCC) tissues/renal tissues; **(D)** A representative IHC staining of “5hmC low” RCC tissue and “5hmC high” RCC tissue. “5hmC low” and “5hmC high” represented cases in which ≤20% and >20% cells were positive for IHC staining of 5hmC, respectively. Scare bar, 200 μm.

To detect the change of 5hmC level in RCC tumorigenesis, we performed DNA dot blot assay using RCC and normal counterparts. The results showed 5hmC was downregulated in 3 RCC tumor samples compared with the matched normal tissues. RCC cell lines also yielded similar results, in which human renal proximal tubular epithelial cell line (HK-2) expressed highest level of 5hmC compared with all four RCC cell lines (Figure 1A). In the following study, we performed IHC staining in RCC and normal kidney tissues adjacent to cancer. IHC staining presented that 5hmC level in 310 RCC patients' tumor tissues was significantly downregulated as compared with that in 248 adjacent kidney tissues (Figure 1B, 1C). With the method mentioned, the cut-off value of 5hmC relative expression level was determined as 20.4% (AUC=0.836, Supplementary Figure S1). So “5hmC low” and “5hmC high” represented cases in which ≤20% and >20% cells were positive for IHC staining of 5hmC, respectively (Figure 1D).

### Patient characteristics

Of 310 RCC patients, those diagnosed with ccRCC were accounted for 230 (74.2%). The median follow-up time was 90.9 months (range 0.3-122.4 months) for all patients. Additionally, 215 (69.4%) patients were followed up for more than 5 years, and 111 (35.8%) patients more than 10 years. During follow-up, 135 (43.5%) patients died and the 5-year and 10-year OS rates were 70.6% and 56.5%, respectively. Table 1 listed the clinicopathological parameters (gender, age, tumor size, capsule invasion, vein invasion, tumor necrosis, TNM stage, clinical stage, Furhman grade and 5hmC level). Briefly, 114 female and 196 male patients with a mean age of 61.8 y (25-87 y). 168 patients identified as were “5hmC low” and 142 were “5hmC high”. Two-sample *t* test and standard nonparametric Mann-Whitney *U*-test showed ccRCC patients in 5hmC low group with a higher clinical stage, higher T stage, lower Furhman grade, compared with 5hmC high group RCC patients (p<0.05, Table 1).

Of 77 RCC patients in the validation cohort, 59 (76.6%) patients were diagnosed with ccRCC. The median follow-up time was 82.1 months (range 1.1-133.6 months). The detailed clinical parameters of enrolled patients in development cohort and validation cohort were presented in Supplementary Table s1, there was no significant difference in clinical parameters between the two cohorts (all p>0.05).

### Correlation analysis between 5hmC expression level and clinicopathological factors of patients with RCC

The correlations between 5hmC and clinicopathological factors were analyzed to define the clinical significance of 5hmC in RCC. Chi-square testing showed that there were no significant correlations between 5hmC level and age, gender, tumor necrosis, N stage, or Furhman grade (Table 2). Remarkably, we found 5hmC level was significantly correlated with tumor size, capsule invasion, vein invasion, T stage, M stage and clinical stage with p values of 0.011, 0.001, 0.012, 0.003, 0.002, 0.001, respectively. Interestingly, these differences were mainly concentrated in other RCC and 5hmC did not seem to affect these clinicopathological variables in ccRCC patients (Table 2). In the validation cohort, chi-square testing also showed the consistent results, which validated our finding (Supplementary Table s2).

### Kaplan-Meier survival analysis between 5hmC expression level and patient survival

**Figure 2:**
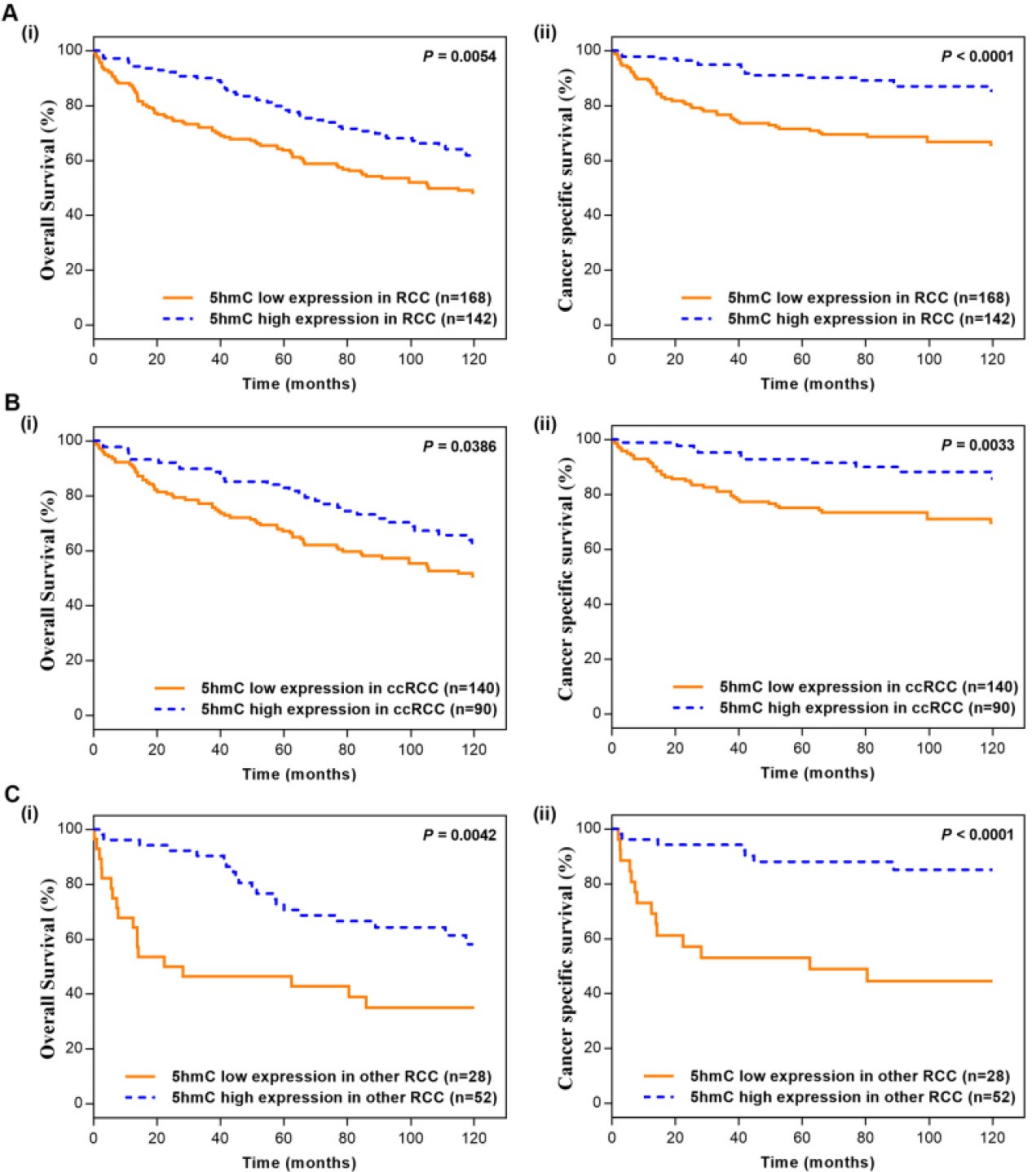
** Kaplan-Meier survival (OS and CSS) curves of RCC patients (A)** RCC patients; **(B)** ccRCC patients;** (C)** other RCC patients. “5hmC low” and “5hmC high” represented cases in which ≤20% and >20% cells were positive for IHC staining of 5hmC, respectively. P values were calculated with the log-rank test.

In the Kaplan-Meier survival analyses, compared to those RCC patients with low 5hmC, patients with high 5hmC had increased OS and CSS (Log-rank, p=0.0054, p<0.0001, respectively, Figure 2A). This finding indicated that high 5hmC expression led to a good prognostic impact for RCC patients. Similar results were also yielded in ccRCC patients (Figure 2B) and other RCC patients (Figure 2C).

### Cox univariate and multivariate analyses of patient survival

Cox univariate and multivariate survival analyses were performed to assess the prognostic value of 5hmC level for RCC and ccRCC patients. Univariate survival analysis showed age, tumor size, capsule invasion, vein invasion, TNM stage, clinical stage, Furhman grade and 5hmC level were factors significantly affecting RCC patient OS. Tumor size, capsule invasion, vein invasion, TNM stage, clinical stage, Furhman grade and 5hmC level were factors significantly affecting CSS. Moreover, multivariate survival analyses revealed that age (HR: 1.043; 95%CI: 1.027-1.059; p<0.001), capsule invasion (HR: 1.745; 95%CI: 1.059-2.877; p=0.029), N stage (HR: 3.110; 95%CI: 1.491-6.485; p=0.002), clinical stage (HR: 1.334; 95%CI: 1.050-1.695; p=0.018), Furhman grade (HR: 1.508; 95%CI: 1.238-1.837; p<0.001) and 5hmC level (HR: 0.657; 95%CI: 0.455-0.949; p=0.025) were independent prognostic factors for RCC patient OS, but only clinical stage (HR: 2.381; 95%CI: 1.892-2.996; p<0.001), Furhman grade (HR: 1.740; 95%CI: 1.325-2.285; p<0.001) and 5hmC level (HR: 0.404; 95%CI: 0.231-0.709; p=0.002) were the independent prognostic factors for RCC patient CSS (Table 3, Table 4, respectively).

The effect of 5hmC in prognosis of ccRCC, the major subtype of RCC, were also of interest. Univariate and multivariate survival analyses revealed that age (HR: 1.042; 95%CI: 1.023-1.061; p<0.001), N stage (HR: 2.176; 95%CI: 0.989-4.786; p=0.053), clinical stage (HR: 1.537; 95%CI: 1.257-1.880; p<0.001) and Furhman grade (HR: 1.349; 95%CI: 1.068-1.703; p=0.012) were independent prognostic factors for ccRCC patient OS, but vein invasion (HR: 2.273; 95%CI: 1.187-4.351; p=0.013), clinical stage (HR: 1.940; 95%CI: 1.385-2.718; p<0.001), Furhman grade (HR: 1.661; 95%CI: 1.179-2.339; p=0.004) and 5hmC level (HR: 0.381; 95%CI: 0.188-0.774; p=0.008) were the independent prognostic factors for ccRCC patient CSS (Table 5, Table 6).

These results suggested that 5hmC level could be considered as an important prognostic biomarker for both RCC and ccRCC.

### Construction of nomogram to predict survival probability

**Figure 3:**
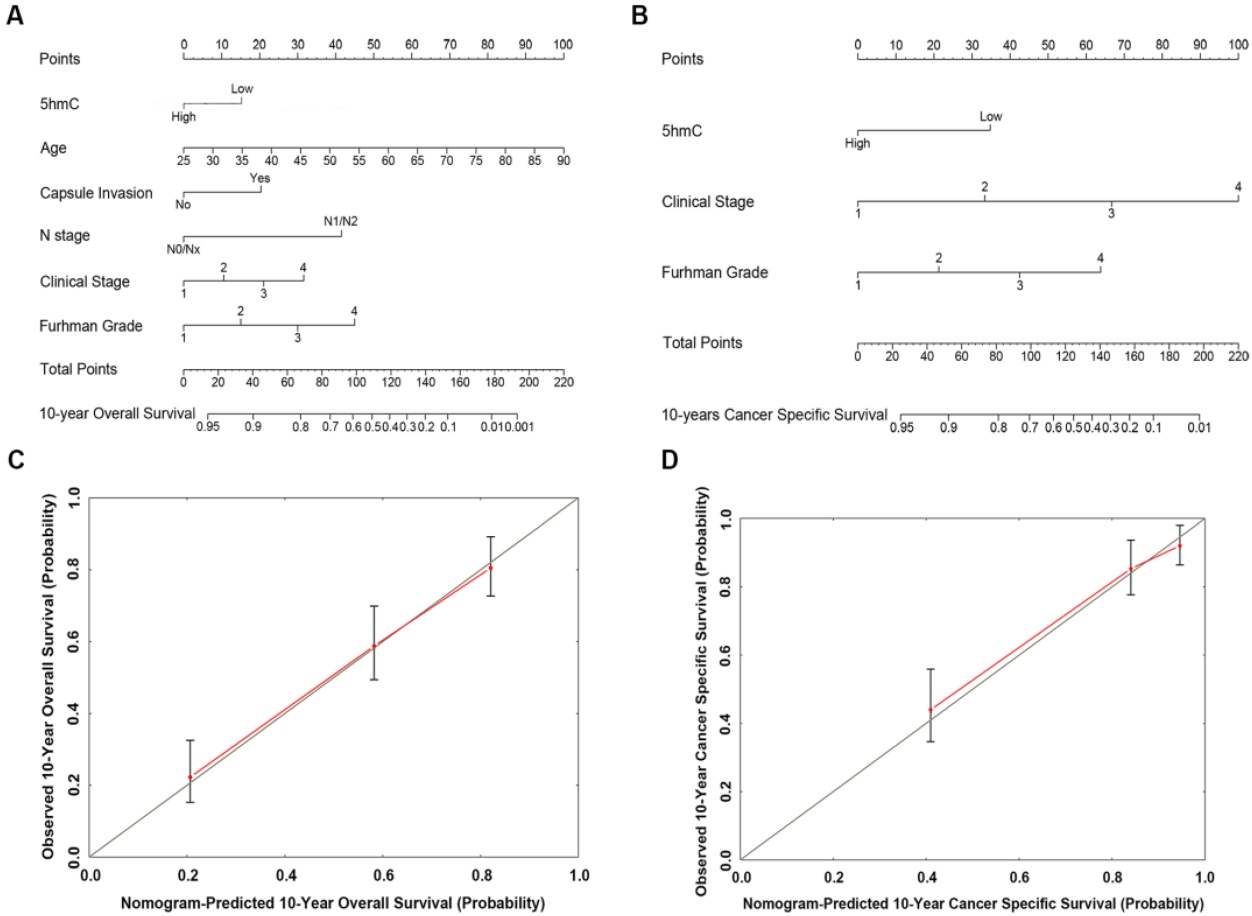
** The predicted nomograms and calibration curves of RCC patient survival (A)** the nomogram developed for OS among 310 RCC patients; **(B)** the nomogram developed for CSS among 310 RCC patients;** (C)** The calibration curve developed for OS among 310 RCC patients;** (D)** The calibration curve developed for CSS among 310 RCC patients. For the nomogram, the points for each variable were calculated by drawing a straight line from a variable value to the axis labelled “Points”. The score sum was converted to a probability in the lowest axis.

**Figure 4:**
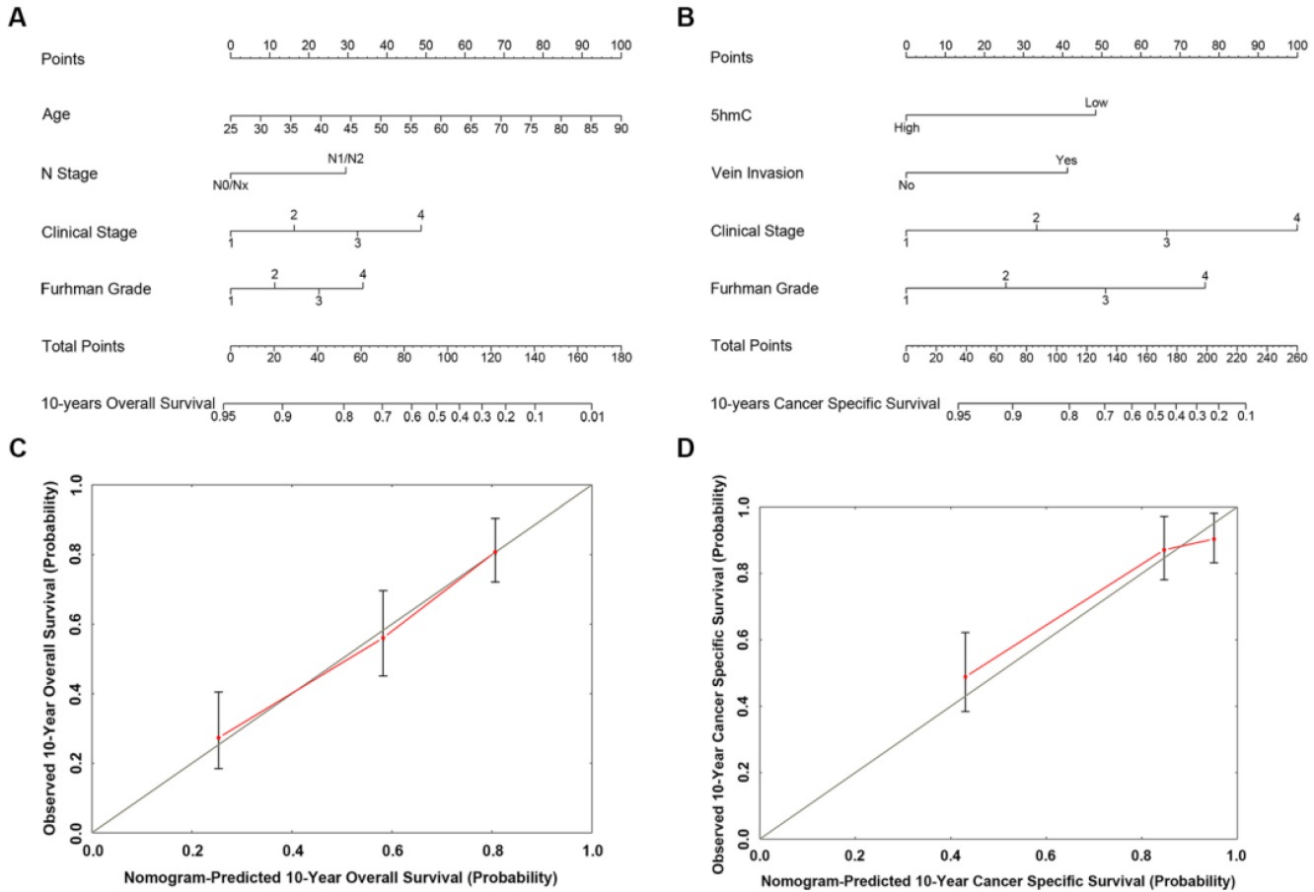
** The predicted nomograms and calibration curves of ccRCC patient survival (A)** The nomogram developed of OS among 230 ccRCC patients; **(B)** The nomogram developed for CSS among 230 ccRCC patients;** (C)** The calibration curve developed for OS among 230 ccRCC patients;** (D)** The calibration curve developed for CSS among 230 ccRCC patients. In the nomogram, the points for each variable were calculated by drawing a straight line from a patient's variable value to the axis labelled “Points”. The score sum was converted to a probability in the lowest axis.

Based on our cox regression analyses, nomograms were constructed to calculate each RCC patient survival probability directly. The 10-year OS (Figure 3A) and CSS (Figure 3B) probabilities of RCC patients were able to be accurately calculated via the nomograms according to the information of each patient (5hmC, age, capsule invasion, N stage, clinical stage, Furhman grade). The calibration curves displayed good agreements of the nomogram-predicted probability with the actual probability for OS (Figure 3C) and CSS (Figure 3D), which indicated that these nomograms had a great value of prediction. Figure 4A and 4B were the nomograms of ccRCC patient 10-year OS and CSS, respectively. The calibration curves shown in Figure 4C and 4D, also displayed good agreement.

### External validation for the prediction nomograms

To confirm the stability of the prediction nomograms, external data validations were performed, which was independently collected in another center (TMA). For 10-year OS prediction of RCC, the sensitivity was 77.3% and the specificity was 81.8%; for 10-year OS prediction of ccRCC, the sensitivity was 76.7% and the specificity was 82.8% (Supplementary Table s3-s4).

Taken together, the results well validated the main findings, the prediction nomograms exhibit high accuracy and stability and are well generalized for other independent datasets.

## Discussion

As a mature epigenetic abnormal change, suppression of 5mC is a common DNA modification in human malignant tumors. It therefore has great potential as a kind of cancer treatment target 16, 23-24. Accumulating evidence suggests that 5hmC confers unique epigenetics, and it can play an important role in multiple tumors, such as liver cancer, melanoma, and acute myeloid leukemia 25-28. In this study, both dot blot and IHC indicated that 5hmC was significantly decreased in RCC compared with those in their normal tissue counterparts. Kaplan-Meier curves revealed that high 5hmC level led to good prognostic impact for RCC patients. Our study established 5hmC loss as a probably common epigenetic characteristic in human tumors including RCC, as with the other reports 25-28. For this reason, we proposed that 5hmC loss might correlated with kidney tumorigenesis and then investigated.

Numerous studies have shown that the loss of 5hmC is associated with the cancer aggressiveness 25-26. In gastric cancer, a decrease in 5hmC promoted the metastasis of gastric cancer cells. Furthermore, suppression of 5hmC was more likely to be present in high grade pathological and large volume tumors in gliomas. In the melanoma, glioma, and esophageal squamous cell carcinoma, the reduction of 5hmC was shown to be an epigenetic hallmark 29-31. However, the prognostic value of 5hmC in renal cell carcinoma (RCC) is still unclear.

This study indicated that low 5hmC level was significantly associated with tumor size, capsule invasion, vein invasion, T stage, M stage, clinical stage, shorter OS and CSS in our RCC patient cohort. Furthermore, Kaplan-Meier curves revealed that the high 5hmC expression had a good prognostic impact in RCC patients. More importantly, cox univariate and multivariate survival analyses further indicated 5hmC was independent prognostic factor for RCC affecting patient survival. These findings suggested that 5hmC might have great values of prognostic in RCC patients.

In recent years, nomogram has been widely used in clinical research modeling. It transforms the complex cox regression survival analyses into a simple and visualized graph, which makes the results of the prediction model more readable and has higher application value. The advantage makes the nomograms get more attention and application in medical research and clinical practice. With the 10-year follow-up data of 310 RCC patients, we developed nomograms to calculate RCC survival probability for the first time. The calibration curves displayed good agreements, which showed nomograms had a great value of prediction. The nomograms may be used to calculate the 10-year OS and CSS probabilities of RCC patients accurately according to the information of each patient. Therefore, clinicians can use these nomograms to make the treatment planning and patient-clinician communication. In addition, our findings were validated with an independent external cohort included 77 RCC cases. The prediction nomograms exhibit high accuracy and stability and are well generalized for other independent datasets.

Of course, considering the effect of racial/ethnic differences and the limitation of small amount of data, multiple center data and more cases are needed for further study.

## Conclusion

5hmC was significantly downregulated in RCC patients. The level of 5hmC was significantly correlated with capsule invasion, vein invasion, T stage, M stage and clinical stage. High 5hmC level led to good prognostic impact for RCC patients and 5hmC was independent prognostic factor in RCC patient survival. In order to establish 5hmC as a novel biomarker for RCC in future, more investigations of its expression, function and regulation are warranted.

## Supplementary Material

Supplementary figures and tables.

## Figures and Tables

**Table 1:** Clinical characteristics of ccRCC and other RCC patients.

Variables	RCC (n=310)	ccRCC (n=230)	other RCC (n = 80)	*p* value
**Age (years), n (%)**				0.156
Average/Median	61.8±13.5/64	61.3±13.5/63	63.5±13.5/66	
Range	25-87	25-87	28-87	
< 65	161 (51.9)	125 (54.3)	36 (45.0)	
≥ 65	149 (48.1)	105 (45.7)	44 (55.0)	
**Gender, n (%)**				0.234
Female	114 (36.8)	89 (38.7)	25 (31.2)	
Male	196 (63.2)	141 (61.3)	55 (68.8)	
**Tumor Size (cm), n (%)**				0.771
Average/Median	6.2±3.7/5.0	6.1±3.6/5.0	6.4±4.1/5.0	
Range	0.5-23.0	0.9-23.0	0.5-18.0	
≤ 5	159 (51.3)	115 (50.0)	44 (55.0)	
> 5	151 (48.7)	115 (50.0)	36 (45.0)	
**Capsule Invasion, n (%)**				0.097
No	226 (72.9)	162 (70.4)	64 (80.0)	
Yes	84 (27.1)	68 (29.6)	16 (20.0)	
**Vein Invasion, n (%)**				0.187
No	252 (81.3)	183 (79.6)	69 (86.2)	
Yes	58 (18.7)	47 (20.4)	11 (13.8)	
**Necrosis, n (%)**				0.044
No	281 (90.6)	213 (92.6)	68 (85.0)	
Yes	29 (9.4)	17 (7.4)	12 (15.0)	
**T Stage, n (%)**				0.035
T1	179 (57.7)	125 (54.3)	54 (67.5)	
T2	25 (8.1)	18 (7.8)	7 (8.8)	
T3	102 (32.9)	85 (37.0)	17 (21.2)	
T4	4 (1.3)	2 (0.9)	2 (2.5)	
**N Stage, n (%)**				0.461
N0/Nx	298 (96.1)	220 (95.7)	78 (97.5)	
N1	6 (1.9)	5 (2.2)	1 (1.3)	
N2	6 (1.9)	5 (2.2)	1 (1.3)	
**M Stage, n (%)**				0.871
M0/Mx	288 (92.9)	214 (93.0)	74 (92.5)	
M1	22 (7.1)	16 (7.0)	6 (7.5)	
**Clinical Stage, n (%)**				0.026
1	175 (56.5)	121 (52.6)	54 (67.5)	
2	25 (8.1)	18 (7.8)	7 (8.8)	
3	85 (27.4)	73 (31.7)	12 (15.0)	
4	25 (8.1)	18 (7.8)	7 (8.8)	
**Furhman Grade, n (%)**				<0.001
1	64 (20.6)	62 (27.0)	2 (2.5)	
2	122 (39.4)	90 (39.1)	32 (40.0)	
3	89 (28.7)	62 (27.0)	27 (33.8)	
4	35 (11.3)	16 (7.0)	19 (23.8)	
**5hmC%, n (%)**				<0.001
Average/Median	28.2±25.0/20	23.8±21.3/20	40.7±30.2/40	
Range	0-23.0	0-90	1-100	
Low (≤20%)	168 (54.2)	140 (60.9)	28 (35.0)	
High (>20%)	142 (45.8)	90 (39.1)	52 (75.0)	

**Table 2:** Correlations between 5hmC expression and clinicopathological factors of patients with RCC and ccRCC.

Characteristics	5hmC expression (RCC)			5hmC expression (ccRCC)			5hmC expression (other RCC)		
	Low (n=168)	High (n=142)	*p*	Low (n=140)	High (n=90)	*p*	Low (n=28)	High (n=52)	*p*
Age (years), n			0.864			0.804			0.451
< 65	88	73		77	48		11	25	
≥ 65	80	69		63	42		17	27	
Gender, n			0.959			0.546			0.527
Female	62	52		52	37		10	15	
Male	106	90		88	53		18	37	
Tumor Size (cm), n			0.011			0.177			0.011
≤ 5	75	84		65	50		10	34	
> 5	93	58		75	40		18	18	
Capsule Invasion, n			0.001			0.285			<0.001
No	110	116		95	67		15	49	
Yes	58	26		45	23		13	3	
Vein Invasion, n			0.012			0.141			0.071
No	128	124		107	76		21	48	
Yes	40	18		33	14		7	4	
Necrosis, n			0.779			0.394			0.646
No	153	128		128	85		25	43	
Yes	15	14		12	5		3	9	
T Stage, n			0.003			0.260			<0.001
T1-T2	98	106		83	60		15	46	
T3-T4	70	36		57	30		13	6	
N Stage, n			0.140			0.349			0.580
N0/Nx	159	139		132	88		27	51	
N1-N2	9	3		8	2		1	1	
M Stage, n			0.002			0.024			0.018
M0/Mx	149	139		126	88		23	51	
M1	19	3		14	2		5	1	
Clinical Stage, n			0.001			0.121			<0.001
1-2	94	106		79	60		15	46	
3-4	74	36		61	30		13	6	
Furhman Grade, n			0.094			0.479			0.602
1-2	108	78		95	57		13	21	
3-4	60	64		45	33		15	31	

**Table 3:** Cox univariate and multivariate analyses of overall survival among 310 RCC patients.

RCC patients (n = 310)	Univariate analysis			Multivariate analysis		
	HR	95% CI	*p* value	HR	95% CI	*p* value
Gender (F/M)	1.082	0.760 - 1.539	0.662	-	-	-
Age (years)	1.043	1.027 - 1.059	< 0.001	1.043	1.027 - 1.059	< 0.001
Tumor Size (cm)	1.104	1.060 - 1.150	< 0.001	1.020	0.966 - 1.078	0.477
Capsule Invasion (yes/no)	3.187	2.260 - 4.494	< 0.001	1.745	1.059 - 2.877	0.029
Vein Invasion (yes/no)	2.840	1.958 - 4.120	< 0.001	1.364	0.788 - 2.362	0.268
Necrosis (yes/no)	1.588	0.941 - 2.680	0.083	-	-	-
T Stage (T3-T4 vs. T1-T2)	3.368	2.396 - 4.734	< 0.001	1.504	0.497 - 3.271	0.760
N Stage (N1-N2 vs. N0/Nx)	4.914	2.626 - 9.198	< 0.001	3.110	1.491 - 6.485	0.002
M Stage (M1 vs. M0/Mx)	4.224	2.584 - 6.904	< 0.001	1.537	0.670 - 3.523	0.310
Clinical Stage	1.783	1.526 - 2.082	< 0.001	1.334	1.050 - 1.695	0.018
Furhman Grade	1.475	1.228 - 1.771	< 0.001	1.508	1.238 - 1.837	< 0.001
5hmC (high/low)	0.624	0.441 - 0.884	0.008	0.657	0.455 - 0.949	0.025

**Table 4:** Cox univariate and multivariate analyses of cancer specific survival among 310 RCC patients.

RCC patients (n = 310)	Univariate analysis			Multivariate analysis		
	HR	95% CI	*p* value	HR	95% CI	*p* value
Gender (F/M)	1.069	0.657 - 1.740	0.788	-	-	-
Age (years)	1.013	0.995 - 1.032	0.163	-	-	-
Tumor Size (cm)	1.166	1.111 - 1.224	< 0.001	1.002	0.935 - 1.073	0.961
Capsule Invasion (yes/no)	5.553	3.449 - 8.941	< 0.001	1.527	0.706 - 3.302	0.282
Vein Invasion (yes/no)	5.662	3.543 - 9.049	< 0.001	1.427	0.736 - 2.764	0.292
Necrosis (yes/no)	1.571	0.779 - 3.165	0.207	-	-	-
T Stage (T3-T4 vs. T1-T2)	7.577	4.469 - 12.847	< 0.001	1.736	0.339 - 8.883	0.508
N Stage (N1-N2 vs. N0/Nx)	7.195	3.650 - 14.184	< 0.001	2.077	0.957 - 4.508	0.064
M Stage (M1 vs. M0/Mx)	6.954	4.001 - 12.088	< 0.001	1.247	0.460 - 3.380	0.665
Clinical Stage	2.693	2.138 - 3.392	< 0.001	2.381	1.892 - 2.996	< 0.001
Furhman Grade	1.708	1.328 - 2.197	< 0.001	1.740	1.325 - 2.285	< 0.001
5hmC (high/low)	0.355	0.208 - 0.606	< 0.001	0.404	0.231 - 0.709	0.002

**Table 5:** Cox univariate and multivariate analyses of overall survival among 230 ccRCC patients.

ccRCC patients (n = 230)	Univariate analysis			Multivariate analysis		
	HR	95% CI	*p* value	HR	95% CI	*p* value
Gender (F/M)	1.058	0.702 - 1.594	0.788	-	-	-
Age (years)	1.044	1.026 - 1.063	< 0.001	1.042	1.023 - 1.061	< 0.001
Tumor Size (cm)	1.092	1.039 - 1.148	0.001	1.016	0.930 - 1.366	0.900
Capsule Invasion (yes/no)	2.886	1.928 - 4.320	< 0.001	1.723	0.865 - 4.168	0.274
Vein Invasion (yes/no)	2.747	1.788 - 4.221	< 0.001	1.700	0.899 - 3.217	0.103
Necrosis (yes/no)	2.010	1.040 - 3.885	0.038	1.153	0.584 - 2.276	0.682
T Stage (T3-T4 vs. T1-T2)	3.070	2.052 - 4.593	< 0.001	1.246	0.790 - 3.016	0.282
N Stage (N1-N2 vs. N0/Nx)	5.112	2.543 - 10.276	< 0.001	2.176	0.989 - 4.786	0.053
M Stage (M1 vs. M0/Mx)	3.886	2.155 - 7.009	< 0.001	1.327	0.478 - 3.686	0.587
Clinical Stage	1.749	1.450 - 2.109	< 0.001	1.537	1.257 - 1.880	< 0.001
Furhman Grade	1.456	1.167 - 1.816	0.001	1.349	1.068 - 1.703	0.012
5hmC (high/low)	0.658	0.430 - 1.009	0.055	0.724	0.462 - 1.133	0.158

**Table 6:** Cox univariate and multivariate analyses of cancer specific survival among 230 ccRCC patients.

ccRCC patients (n = 230)	Univariate analysis			Multivariate analysis		
	HR	95% CI	*p* value	HR	95% CI	*p* value
Gender (F/M)	1.228	0.684 - 2.204	0.493	-	-	-
Age (years)	1.012	0.991 - 1.034	0.269	-	-	-
Tumor Size (cm)	1.142	1.074 - 1.214	< 0.001	1.072	0.895 - 1.455	0.497
Capsule Invasion (yes/no)	4.166	2.377 - 7.302	< 0.001	1.390	0.914 - 4.633	0.146
Vein Invasion (yes/no)	5.811	3.326 - 10.155	< 0.001	2.273	1.187 - 4.351	0.013
Necrosis (yes/no)	1.680	0.665 - 4.247	0.273	-	-	-
T Stage (T3-T4 vs. T1-T2)	5.735	3.086 - 10.658	< 0.001	1.163	0.742 - 3.472	0.876
N Stage (N1-N2 vs. N0/Nx)	7.653	3.544 - 16.525	< 0.001	2.203	0.893 - 5.434	0.086
M Stage (M1 vs. M0/Mx)	6.206	3.153 - 12.216	< 0.001	2.129	0.645 - 7.026	0.215
Clinical Stage	2.464	1.859 - 3.265	< 0.001	1.940	1.385 - 2.718	< 0.001
Furhman Grade	1.601	1.183 - 2.169	0.002	1.661	1.179 - 2.339	0.004
5hmC (high/low)	0.405	0.207 - 0.791	0.008	0.381	0.188 - 0.774	0.008

## Abbreviations

AUC: Areas Under the Curve
ccRCC: Clear Cell Renal Cell Carcinoma
index: Concordance Index
CSS: Cancer Specific Survival
FBS: Fetal Bovine Serum
5hmC: 5-hydroxymethylcytosine
5mC: DNA Methylation of Cytosine
IDH1/2: Isocitrate Dehydrogenase 1 and 2
IHC: Immunohistochemical
OS: Overall Survival
RCC: Renal Cell Carcinoma
ROC: Receiver Operating Characteristic
RT: Room Temperature
TET: Ten-Eleven-Translocation
VHL: Von Hippel-Lindau
