# Supplementary material for: Prognostic value of downregulated 5-hydroxymethylcytosine expression in renal cell carcinoma: a 10 year follow-up retrospective study
Source: J Cancer. 2020 Jan 1;11(5):1212–22. doi: 10.7150/jca.38283 (PMC6959072; doi:10.7150/jca.38283)
Supplement: Supplementary file 1 — Supplementary figures and tables. [file jcav11p1212s1.pdf]

Supplementary Figure

Supplementary Figure S1. The cut-off value of 5hmC relative expression level in the ROC curve

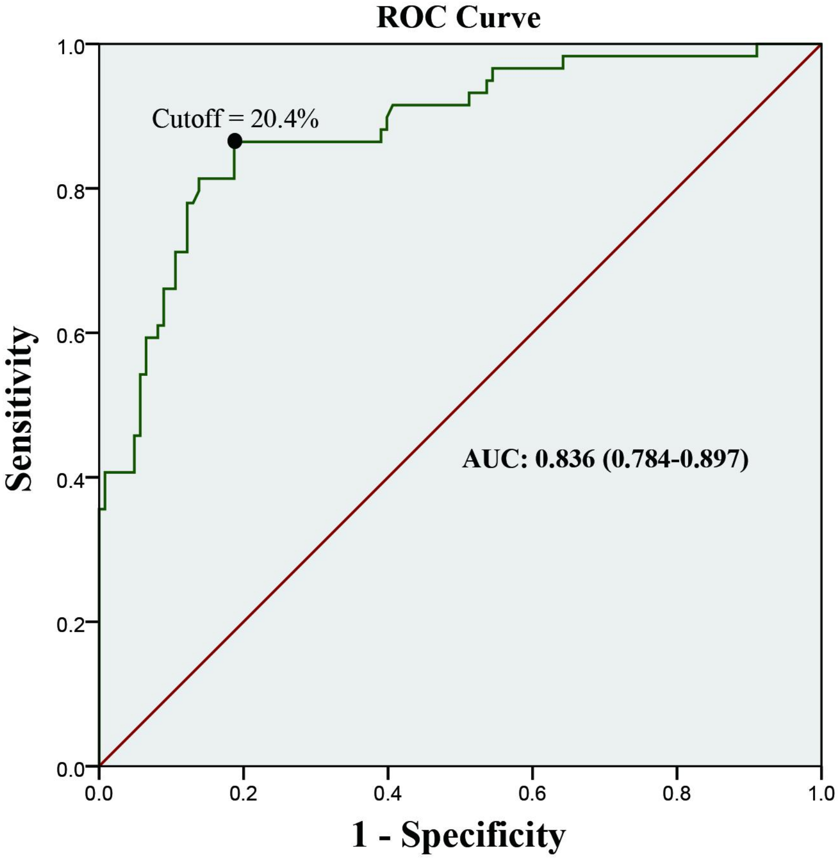

Supplementary Table s1. Clinical characteristics of enrolled patients in development cohort and validation cohort.

Variables	Development cohort (n=310)	Validation cohort (n=77)	*p* value
**Age (years), n (%)**			0.837
Average/Median	61.8±13.5/64	60.9±14.8/63	
< 65	161 (51.9)	41 (53.2)	
≥ 65	149 (48.1)	36 (46.8)	
**Gender, n (%)**			0.481
Female	114 (36.8)	25 (32.5)	
Male	196 (63.2)	52 (67.5)	
**Tumor Size (cm), n (%)**			0.758
Average/Median	6.2±3.7/5.0	6.0±3.8/5.1	
≤ 5	159 (51.3)	41 (53.2)	
> 5	151 (48.7)	36 (46.8)	
**Capsule Invasion, n (%)**			0.246
No	226 (72.9)	51 (66.2)	
Yes	84 (27.1)	26 (33.8)	
**Vein Invasion, n (%)**			0.095
No	252 (81.3)	56 (72.7)	
Yes	58 (18.7)	21 (27.3)	
**T Stage, n (%)**			0.775
T1	179 (57.7)	47 (61.0)	
T2	25 (8.1)	5 (6.5)	
T3	102 (32.9)	22 (28.6)	
T4	4 (1.3)	3 (3.9)	
**N Stage, n (%)**			0.140
N0/Nx	298 (96.1)	73 (94.8)	
N1	6 (1.9)	3 (3.9)	
N2	6 (1.9)	1 (1.3)	
**M Stage, n (%)**			0.840
M0/Mx	288 (92.9)	73 (94.8)	
M1	22 (7.1)	4 (5.2)	
**Clinical Stage, n (%)**			0.901
1	175 (56.5)	42 (54.5)	
2	25 (8.1)	7 (9.1)	
3	85 (27.4)	23 (29.9)	
4	25 (8.1)	5 (6.5)	
**Furhman Grade, n (%)**			0.652
1	64 (20.6)	17 (22.1)	
2	122 (39.4)	29 (37.7)	
3	89 (28.7)	24 (31.2)	
4	35 (11.3)	7 (9.1)	
**5hmC%, n (%)**			0.577
Average/Median	28.2±25.0/20	30.6±23.1/22	
Low (≤20%)	168 (54.2)	39 (50.6)	
High (>20%)	142 (45.8)	38 (49.4)	

Supplementary table s2. Correlations between 5hmC expression and clinicopathological factors of RCC patients in validation cohort.

Characteristics	5hmC expression (RCC)		
	Low (n=39)	High (n=38)	*p* value
**Age (years), n**			0.742
< 65	23	21	
≥ 65	16	17	
**Gender, n**			0.501
Female	13	10	
Male	26	28	
**Tumor Size (cm), n**			0.050
≤ 5	17	25	
> 5	22	13	
**Capsule Invasion, n**			0.044
No	25	32	
Yes	14	6	
**Vein Invasion, n**			0.050
No	28	34	
Yes	11	4	
**T Stage, n**			0.020
T1-T2	21	30	
T3-T4	18	8	
**N Stage, n**			0.571
N0/Nx	37	37	
N1-N2	2	1	
**M Stage, n**			0.028
M0/Mx	32	37	
M1	7	1	
**Clinical Stage, n**			0.022
1-2	20	29	
3-4	19	9	
**Furhman Grade, n**			0.285
1-2	21	25	
3-4	18	13	

Supplementary table s3. External data validation of the OS prediction of RCC.

		10-year OS (Validation cohort)		Total
		Alive	Dead	
10-year OS (Prediction nomogram)	Alive	34	10	44
	Dead	6	27	33
Total		40	37	77

Sensitivity =34/(34+10)=77.3%

Specificity =27/(27+6)=81.8%

Supplementary table s4. External data validation of the OS prediction of ccRCC.

		10-year OS (Validation cohort)		Total
		Alive	Dead	
10-year OS (Prediction nomogram)	Alive	23	7	30
	Dead	5	24	29
Total		28	31	59

Sensitivity =23/(23+7)=76.7%

Specificity =24/(24+5)=82.8%
